# Changes in Specific Biomarkers Indicate Cardiac Adaptive and Anti-inflammatory Response of Repeated Recreational SCUBA Diving

**DOI:** 10.3389/fcvm.2022.855682

**Published:** 2022-03-14

**Authors:** Jerka Dumić, Ana Cvetko, Irena Abramović, Sandra Šupraha Goreta, Antonija Perović, Marina Njire Bratičević, Domagoj Kifer, Nino Sinčić, Olga Gornik, Marko Žarak

**Affiliations:** ^1^Department of Biochemistry and Molecular Biology, University of Zagreb Faculty of Pharmacy and Biochemistry, Zagreb, Croatia; ^2^Department of Medical Biology, University of Zagreb School of Medicine, Zagreb, Croatia; ^3^Department of Laboratory Diagnostics, Dubrovnik General Hospital, Dubrovnik, Croatia; ^4^Department of Biophysics, University of Zagreb Faculty of Pharmacy and Biochemistry, Zagreb, Croatia; ^5^Clinical Department of Laboratory Diagnostics, Dubrava University Hospital, Zagreb, Croatia

**Keywords:** recreational SCUBA (rSCUBA) diving, copeptin (CPP), neutrophil-to-lymphocyte ration (NLR), immunoglobulin, complement C3 and C4, N-glycosylation, cell-free DNA

## Abstract

**Objective:**

Recreational SCUBA (rSCUBA) diving has become a highly popular and widespread sport. Yet, information on molecular events underlying (patho)physiological events that follow exposure to the specific environmental conditions (hyperbaric conditions, coldness, immersion, and elevated breathing pressure), in which rSCUBA diving is performed, remain largely unknown. Our previous study suggested that repeated rSCUBA diving triggers an adaptive response of cardiovascular and immune system. To elucidate further molecular events underlying cardiac and immune system adaptation and to exclude possible adverse effects we measured blood levels of specific cardiac and inflammation markers.

**Methods:**

This longitudinal intervention study included fourteen recreational divers who performed five dives, one per week, on the depth 20–30 m that lasted 30 min, after the non-dive period of 5 months. Blood samples were taken immediately before and after the first, third, and fifth dives. Copeptin, immunoglobulins A, G and M, complement components C3 and C4, and differential blood count parameters, including neutrophil-to-lymphocyte ratio (NLR) were determined using standard laboratory methods. Cell-free DNA was measured by qPCR analysis and N-glycans released from IgG and total plasma proteins (TPP), were analyzed by hydrophilic interaction ultra-performance liquid chromatography.

**Results:**

Copeptin level increased after the first dive but decreased after the third and fifth dive. Increases in immunoglobulins level after every dive and during whole studied period were observed, but no changes in C3, C4, and cfDNA level were detected. NLR increased only after the first dive. IgG and TPP N-glycosylation alterations toward anti-inflammatory status over whole studied period were manifested as an increase in monogalyctosylated and core-fucosylated IgG N-glycans and decrease in agalactosylated TPP N-glycans.

**Conclusion:**

rSCUBA diving practiced on a regular basis promotes anti-inflammatory status thus contributing cardioprotection and conferring multiple health benefits.

## Introduction

Recreational SCUBA diving [diving up to the depth of 40 m with direct access to the surface, without decompression stop, using compressed air or nitrox (a mixture of oxygen and nitrogen where the oxygen content does not exceed 40%) as a breathing gas (1)] has become a highly popular and widespread sport over the past few decades. Although practicing rSCUBA diving is relatively safe and rarely results in fatalities, its effects on human health, either acute or chronic, remain largely unknown (2), mainly due to the relatively small number of scientific studies investigating its (patho)physiological effects at the molecular levels. Furthermore, even if only studies related to rSCUBA diving are considered, omitting those on technical SCUBA diving, the results are often incomparable due to the differences in studies’ designs (e.g., depth, duration, type of breathing gas, and frequency of dives, anthropological proprieties, physical and overall health conditions of divers, their experience, and level of training).

We have recently undertaken a longitudinal intervention study that comprised fourteen divers who performed five dives, one per week on the depth 20–30 m that lasted 30 min, after a non-dive period of 5 months. The results so far suggested the activation of adaptive mechanisms of the cardiovascular (CV) and immune systems, muscles, and hematopoiesis (3, 4). Yet, some ambiguous results related to hs-CRP, proposed as an independent predictor of CV disease development (5, 6), prompt us to expend the study by introducing some other cardiac and inflammatory markers, aiming to further elucidate molecular events triggered by repeated rSCUBA diving.

Copeptin (CPP), a 39-amino acids long glycopeptide, is a specific neurohormonal and cardiac marker that has predictive value for the future CV diseases development. It is a C-terminal part of the pre-pro-arginine vasopressin (pre-proAVP) precursor (CT-proAVP) (7), thus mirroring AVP concentration and being used as a surrogate marker of AVP (8, 9). AVP, also known as an antidiuretic hormone, is a vasoactive pituitary hormone, whose primary function is water regulation and homeostasis of electrolytes. Its release is mainly stimulated by hyperosmolarity but also by numerous non-specific factors such as hypoxia (10) that also occurs during rSCUBA diving (11). Furthermore, immersion, one of the major physical phenomena that follows rSCUBA diving, induces diuresis and natriuresis with consequent increase of free water clearance (12) enabled precisely by a decrease of AVP blood concentration (13). In diving conditions, the decrease of AVP concentration is not affected only and primarily by plasma osmolarity changes, but also by the altered activation of cardiac and aortic arch baroreceptors (14). If the organism is exposed to endogenous stress caused by extreme rSCUBA diving conditions, AVP system can be activated and CPP is released into circulation physiologically and regardless of any significant necrosis of cardiac cells. Also, changed filling of left ventricle, due to alteration of body fluid balance and cardiac output, stimulates cardiac baroreceptors and activates renin-angiotensin-aldosterone system (RAAS), which all together subsequently leads to CPP secretion from posterior pituitary gland (15). In addition to hyperosmolarity, AVP release can be induced by several non-osmotic stimuli, such as hypovolemia, pain, nausea, and certain drugs (16), but also through “immunoneuroendocrine interface” with interleukin-6 (IL-6) as a key player (17). It was also shown that AVP induces IL-6 production of neonatal rat cardiac fibroblasts by activating V1A receptor signaling *via* a G protein-coupled receptor kinase 2/nuclear factor -kappa B (GRK2/NF-κB) pathway (18). In the recent years, CPP has been studied as a diagnostic and prognostic marker in cardiac diseases such as acute myocardial infarction and heart failure (19), so we consider it as a suitable marker for further elucidation of the effects of rSCUBA diving on the CV system.

Stretching and overload of cardiomyocytes may result in cardiac microdamage, which can be repaired by activation of immune system and triggering local inflammation. Continuous cycles of damage and repair in cardiac tissue result in latent, subclinical inflammation and consequently, in changes in concentrations of various cellular and humoral markers of inflammation in circulation.

Neutrophil-to-lymphocyte ratio (NLR), an established inflammation marker, reflects the intensity of immune/inflammatory reaction and (patho)physiological response to stressor or disease and may provide information on the balance between innate and adaptive immune responses. It has been proposed as a prognostic marker of development of metabolic syndrome (20), atherosclerosis (21), cardiac diseases (22, 23) as well as different cancers (24–26). Physical activity and exercise are also followed by alterations in NLR, which are direct result of changes in count of neutrophils and lymphocytes, which are a part of the complex response of cellular immune system to various stimuli coming from autonomic nervous system, endocrine system and circulating mediators (27). Exercise-induced increase in cardiac output and blood flow triggers leukocyte demargination from the vascular, hepatic, spleen, and pulmonary reservoirs.

Besides immune cells, enhanced blood flow results in redeployment of humoral immune components, such as immunoglobulins, complement components, and cytokines, thus boosting immunosurveillance. Still, the effects of exercise on immunoglobulins, besides secretory IgA (sIgA) (28), have not been systematically studied, whereas information on impact of rSCUBA diving on serum level on Ig does not exist in available scientific literature. Several studies showed increased levels of certain immunoglobulin classes after moderate exercise (29–31), suggesting positive effects on the immune system. Yet, the relation between serum concentrations of total Igs or certain Ig classes and different cardiovascular diseases is not yet fully resolved (32–34), but most of the studies suggest cardioprotective effect of higher concentrations of Ig (within the corresponding reference range). It was also speculated that a low level of total Igs is a cardiovascular risk factor, namely that Igs have a protective function in the cardiovascular system (35, 36).

The complement system, an immune effector system, is usually linked to the innate immunity reactions, but in the recent years its role in metabolic and cardiovascular events has been recognized. In this sense, particularly interesting are complement components C3 and C4 because they have been associated with cardiovascular risk factors (37, 38), diabetes (39), and metabolic syndrome (40) that was also linked with increase in C4 level (41). Although scientific data on effects of exercise on C3 and C4 level are not consistent (29–31, 42–45), many of them suggest that negative correlation between C3 and C4 levels with general health (43, 46, 47).

N-glycosylation is one of the most abundant posttranslational modifications. N-glycans significantly modulate biological functions of the glycoproteins on which they are attached. For instance, changes in N-glycan chains attached to the constant Fc domain of IgG molecule can modulate conformation, stability, and half-life of IgG molecule, but even more importantly, its affinity for Fc receptors, thus making it more, or less inflammatory (48–52). In the recent years, N-glycosylation has been recognized as an important phenotypic feature of many pathological processes and conditions, such as chronic inflammatory and autoimmune diseases (53, 54) as well as diabetes (55), cardiovascular disease risk and subclinical atherosclerosis (56–59), and many others. Yet, there are only few studies on the effects of exercise on N-glycosylation profiles of TPP and IgG, whereas the effects of rSCUBA diving on these biomarkers have not been studied so far. One of these studies showed that intense anaerobic physical exercise induces anti-inflammatory changes in IgG N-glycosylation profile (60), whereas studies of exercise impact on level of GlycA, a glycan marker of systemic inflammation, found its decreased levels after exercise (61, 62).

Cell-free DNA are small fragments of DNA, released in circulation from normal cells, in healthy individuals mostly form hematopoietic cells. In certain physiological (e.g., pregnancy, exercise, and aging) and many pathological conditions, its plasma concentration is significantly higher due to the release from damaged or dying cells of affected tissues. Therefore, cfDNA has become a promising biomarker in diagnosis and prognosis of cardiovascular and neurological diseases, diabetes, and cancer, but also an important marker for overtraining syndrome or performance diagnostics. Many studies observed increase in cfDNA concentration after intensive exercises such as different forms of running (63–65), strength training (66, 67), rowing (68), and cycling (69). The cfDNA increase in circulation after exercise has been mainly attributed to the myocyte membrane damage and its increased permeability (64, 70), but also increased leukocytes activity including apoptosis (64) and formation of intravascular neutrophil extracellular traps (71), as well as cellular necrosis (66). All these events may occur during rSCUBA diving, so one could presume an increase in cfDNA level, but it has not been studied so far. cfDNA integrity index (CFI), a ratio of longer to shorter fragments of cfDNA, most often calculated by quantifying the expression of LINE-1 element may provide information on cell death type causing cfDNA increase. Namely, longer cfDNA fragments are usually excreted from necrotic cells, while shorter ones are being released *via* apoptosis (72), so higher CFI is considered an indicator of necrotic processes in the organism.

Therefore, to further elucidate molecular events underlying adaptation processes of cardiovascular and immune system to the repeated rSCUBA diving, in this work we studied levels of copeptin, complement components C3 and C4, certain Ig classes (IgA, IgG, and IgM), and cfDNA, as well as neutrophil-to-lymphocyte ration (NLR) and N-glycosylation profiles of TPP and IgG.

## Materials and Methods

### Participants

The subjects in this longitudinal intervention study were fourteen male recreational divers with a median age (range) of 42 (19–54) years, 3–20 years of diving experience, and fewer than 30 dives per year. The divers were not professional athletes and had not dived for at least 5 months (during the winter period). All divers had a valid diving medical certificate, and none of them had any acute or chronic illness symptoms, which were ruled out by extensive anamnestic data and laboratory tests before the research. The subjects were told about the study’s objectives and standards before giving their signed consent to participate. The research was designed and carried out in accordance with the Helsinki Declaration and was approved by the Ethical Committee of the University of Zagreb Faculty of Pharmacy and Biochemistry.

### Study Design

The study was designed as a cumulative effect study consisting of five experimental dives performed once per week after 5 non-diving months at the Adriatic coast in the period from March to April 2019. The first dive was performed at a maximum depth of 20 m for a total of 30 min, and the remaining four at a maximum depth of 30 m for a total of 30 min. All five dives took place at the same time in the morning, with progressive immersion to the maximum depth (descending velocity of 10 m/min) and gradual ascent to the surface without a decompression stop (ascending velocity of 9 m/min). Wetsuits, dive computers, and open-circuit SCUBA diving devices with compressed air were used as a diving equipment. The sea temperature was between 12 and 15 °C and the air temperature was between 16 and 20 °C.

### Blood Sampling

Six blood samples were obtained from each subject immediately before (pre-dive) and immediately after (post-dive) the first, third, and fifth dives for the time-course evaluation. Blood samples were taken from patients’ antecubital veins straight into vacuum tubes containing K_2_EDTA and clot activator. Differential blood count (DBC) parameters were determined within 1 hour after sampling, as well as the separation of serum and plasma samples, which were then stored at −80 °C until analyses.

### Routine Laboratory Parameters Determination

Routine laboratory parameters were assessed using certified reagents and techniques with appropriate analytical properties in accredited clinical laboratories. Copeptin (CPP) serum concentration was determined using competitive ELISA principle by Human CPP (Copeptin) ELISA kit (Novus Biologicals, LLC, Centennial, CO, USA; Bio-Techne Ltd., Oxford, United Kingdom) on the automated ELISA analyzer Siemens BEP 2000 Advance (Siemens Healthcare Diagnostics Walpole, NJ, USA). Immunoglobulin A (IgA), G (IgG), and M (IgM), as well as complement component C3 (C3) and C4 (C4) serum concentrations were measured by immunoturbidimetric assay on Beckman Coulter DxC 700 AU analyzer (Beckman Coulter, Brea, CA, USA). DBC parameters (absolute number of neutrophilic granulocytes and lymphocytes) were determined using Abbott Cell Dyn Ruby (Abbott Diagnostics, Abbott Park, IL, USA) hematology analyzer in K_2_EDTA whole blood samples within 1 h after sampling. Neutrophil-to-lymphocyte ratio (NLR) was calculated by dividing the absolute number of neutrophils with the absolute number of lymphocytes.

### IgG and Total Plasma Proteins N-Glycans Determination

#### Human Plasma IgG Isolation Using Monolithic Protein G Plate

Isolation of IgG from human plasma samples was performed using affinity chromatography as described in a previous publication (74). In short, starting from 100 μL of human plasma, IgG was isolated in a high-throughput analysis using 96-well protein G monolithic plates (BIA Separations, Ajdovščina, Slovenia). Human plasma was diluted 7x with phosphate buffered saline (PBS), applied to monolithic protein G plate, and washed with PBS. IgG elution from monolithic protein G plate was performed using 0.1 M formic acid (Merck, Germany), followed by immediate neutralization using 1 M ammonium bicarbonate (Acros Organics, New Jersey, USA).

#### Release of N-Glycans From IgG and Total Plasma Proteins

IgG elution from human plasma was followed by drying in vacuum centrifuge with the purpose of concentrating the samples. Addition of 30 μL of 1.33% SDS (w/v, Invitrogen, Waltham, USA) was used for IgG denaturation, followed by 10 min incubation at 65 °C. Regarding human plasma samples, starting volume of 10 μL was denatured with 20 μL of 2% SDS (w/v, Invitrogen, USA), followed by 10 min incubation at 65 °C. Following steps in N-glycan release method were the same for both IgG and human plasma samples. Sample denaturation was followed by addition of 10 μL of 4% Igepal-CA630 (v/v, Sigma Aldrich, St. Louis, USA) and 15 min sample mixing on a plate shaker (GFL, Burgwedel, Germany) with the purpose of neutralizing excess SDS. N-glycans were released from IgG/human plasma proteins using 1.2 U of PNGase F (Promega, Madison, USA), followed by overnight incubation at 37 °C.

#### Labeling and Clean-Up of Released N-Glycans

Labeling mixture for released N-glycans was made from 2-aminobenzamide, a fluorescent dye (19.2 mg/mL; Sigma Aldrich, USA), and 2-picoline borane, reductive agent (44.8 mg/mL; Sigma Aldrich, USA) in dimethyl sulfoxide (Sigma Aldrich, USA) and glacial acetic acid (Merck, Germany) mixture (70:30 v/v). Samples were incubated with the fluorescent labeling mixture for 2 h at 65 °C, after which hydrophilic interaction liquid chromatography solid-phase extraction was performed on 0.2 μm GHP filter plate (Pall Corporation, New York, NY, USA) to remove any residuals from samples containing now fluorescently labeled, released N-glycans. After clean-up, N-glycans were eluted using ultra-pure water. Samples containing eluted, fluorescently labeled, released N-glycans were stored at −20 °C until further usage.

#### Analysis of N-Glycans by Hydrophilic Interaction Ultra-Performance Liquid Chromatography

**FIGURE 1:**
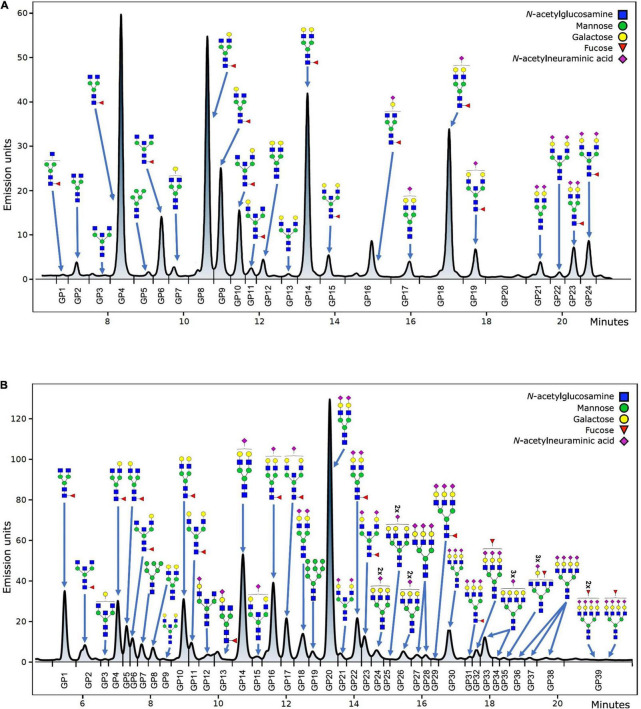
Example of IgG **(A)** and total plasma proteins **(B)** N-glycan chromatogram with the most abundant structures shown for each glycan peak.

Hydrophilic interaction based liquid chromatography was performed on Acquity UPLC H-Class instrument (Waters, Milford, USA) with the purpose of fluorescently labeled N-glycans separation. Empower 3 software, build 3471 (Waters, Milford, MA, USA) was used for instrument control, monitoring separation method, and samples integration. N-glycans separation was done on Waters BEH (bridged ethylene hybrid) Glycan chromatography column. Solvent A was 100 mM ammonium formate, pH 4.4, while acetonitrile (LC-MS grade) was used as solvent B. Separation parameters for IgG N-glycans were as follows: linear gradient of 75–62% acetonitrile at flow rate of 0.4 mL/min in a 27 min analytical run. Separation parameters for human plasma protein N-glycans were as follows: linear gradient of 70–53% acetonitrile at flow rate of 0.56 mL/min in a 25 min analytical run. System was calibrated using hydrolyzed, 2-aminobenzamide labeled glucose oligomers, and individual glycan retention times were transferred to glucose units. Data processing was performed by automatic processing method with a traditional integration algorithm, followed by manual correction of each sample chromatogram, in order to have the same integration pattern for all samples. IgG N-glycans chromatograms separated into 24 glycan peaks (GP1-GP24) (Figure 1A), and human plasma protein N-glycans chromatogram separated into 39 glycan peaks (GP1-GP39) (Figure 1B). Elution time of glycan peaks in each chromatogram was used as a basis for their analysis, measured in glucose units, and followed by comparison to the reference values in the ‘‘GlycoStore’’ database^1^ with the purpose of structural assignment (75, 76). Glycan amount in each glycan peak was expressed as total integrated area percentage. Apart from directly measured glycan traits (24 glycan peaks for IgG N-glycans, and 39 glycan peaks for human plasma protein N-glycans), derived traits were also calculated. Derived traits average various glycosylation features, such as antennary fucosylation, sialylation, level of branching, etc. Description of derived traits are available in Table 1.

**TABLE 1:** Abbreviations, description and calculation formula for derived glycan traits calculated from directly measured glycan traits for IgG N-glycans and total plasma proteins N-glycans.

IgG N-glycans			
**Derived trait**		**Description**	**Formula**
iG0	Agalactosylation	Amount of agalactosylated structures in the total IgG N-glycome	GP1 + GP2 + GP3 + GP4 + GP5 + GP6
iG1	Monogalactosylation	Amount of monogalactosylated structures in the total IgG N-glycome	GP7 + GP8 + GP9 + GP10 + GP11 + GP16
iG2	Digalactosylation	Amount of digalactosylated structures in the total IgG N-glycome	GP12 + GP13 + GP14 + GP15 + GP17 + GP18 + GP19 + GP20 + GP21 + GP22 + GP23 + GP24
iS0	Neutral glycans	Amount of asialylated structures in the total IgG N-glycome	GP1 + GP2 + GP3 + GP4 + GP5 + GP6 + GP7 + GP8 + GP9 + GP10 + GP11 + GP12 + GP13 + GP14 + GP15
iS1	Monosialylation	Amount of monosialylated structures in the total IgG N-glycome	GP16 + GP17 + GP18 + GP19
iS2	Disialylation	Amount of disialylated structures in the total IgG N-glycome	GP20 + GP21 + GP22 + GP23 + GP24
iB	Incidence of bisecting GlcNAc	Amount of structures containing bisecting GlcNAc in the total IgG N-glycome	GP6 + GP10 + GP11 + GP13 + GP15 + GP19 + GP22 + GP24
iCF	Core fucosylation	Amount of structures containing core fucose in the total IgG N-glycome	GP1 + GP4 + GP6 + GP8 + GP9 + GP10 + GP11 + GP14 + GP15 + GP16 + GP18 + GP19 + GP23 + GP24
iOM	Oligomannose glycans	Amount of oligomannose structures in the total IgG N-glycome	GP5

**Total plasma proteins N-glycans**			
LB	Low branching (mono- and biantennary glycans)	Amount of mono- and biantennary structures in the total plasma N-glycome	GP1 + GP2 + GP3 + GP4 + GP5 + GP6 + GP7 + GP8 + GP9 + GP10 + GP11 + GP12 + GP13 + GP14 + GP15 + GP16 + GP17 + GP18 + GP19 + GP20 + GP21 + GP22 + GP23
HB	High branching (tri- and tetraantennary glycans)	Amount of tri- and tetraantennary structures in the total plasma N-glycome	GP24 + GP25 + GP26 + GP27 + GP28 + GP29 + GP30 + GP31 + GP32 + GP33 + GP34 + GP35 + GP36 + GP37 + GP38 + GP39
G0	Agalactosylation	Amount of agalactosylated structures in the total plasma N-glycome	GP1 + GP2 + GP7 + GP19
G1	Monogalactosylation	Amount of monogalactosylated structures in the total plasma N-glycome	GP3 + GP4 + GP5 + GP6 + GP13
G2	Digalactosylation	Amount of digalactosylated structures in the total plasma N-glycome	GP8 + GP9 + GP10 + GP11 + GP12 + GP14 + GP15 + GP16 + GP17 + GP18 + GP20 + GP21 + GP22 + GP23
G3	Trigalactosylation	Amount of trigalactosylated structures in the total plasma N-glycome	GP24 + GP25 + GP26 + GP27 + GP28 + GP29 + GP30 + GP31 + GP32 + GP33 + GP34 + GP35
G4	Tetragalactosylation	Amount of tetragalactosylated structures in the total plasma N-glycome	GP36 + GP37 + GP38 + GP39
S0	Neutral glycans	Amount of asialylated structures in the total plasma N-glycome	GP1 + GP2 + GP3 + GP4 + GP5 + GP6 + GP7 + GP8 + GP9 + GP10 + GP11 + GP19
S1	Monosialylation	Amount of monosialylated structures in the total plasma N-glycome	GP12 + GP13 + GP14 + GP15 + GP16 + GP17
S2	Disialylation	Amount of disialylated structures in the total plasma N-glycome	GP18 + GP20 + GP21 + GP22 + GP23 + GP24 + GP25 + GP26 + GP27
S3	Trisialylation	Amount of trisialylated structures in the total plasma N-glycome	GP28 + GP29 + GP30 + GP31 + GP32 + GP33 + GP34 + GP35 + GP36
S4	Tetrasialylation	Amount of tetrasialylated structures in the total plasma N-glycome	GP37 + GP38 + GP39
CF	Core fucosylation	Amount of structures containing core fucose in the total plasma N-glycome	GP1 + GP2 + GP4 + GP5 + GP6 + GP10 + GP11 + GP13 + GP16 + GP17 + GP22 + GP23 + GP31 + GP34 + GP35
AF	Antennary fucosylation	Amount of structures containing antennary fucose in the total plasma N-glycome	GP27 + GP33 + GP35 + GP39

### Cell-Free DNA i Cell-Free DNA Integrity Determination

Cell-free DNA was isolated from 500 μL of blood plasma using Macherey-Nagel’s (Allentown, USA) NucleoSpin cfDNA XS according to the manufacturer’s instructions. The concentration and the integrity of cfDNA were subsequently determined by qPCR with primers targeting the second open reading frame of the human LINE-1 element. Primers for shorter amplicon of 82-bp LINE-1 (F-primer sequence: 5′-TCACTCAAAGCCGCTCAACTAC-3′; R-primer sequence: 5′-TCTGCCTTCATTTCGTTATGTACC-3′) region were used to quantify total cfDNA (77), while ones for longer amplicon of 224-bp (F-primer sequence: 5′-TCTGCCTTCATTTCGTTATGTACC-3′; R-primer sequence: 5′-TCAGCACCACACCACACCTATTC-3′) were used to calculate the cell-free DNA integrity index (CFI), the ratio of longer cfDNA fragments to the shorter ones (78) to estimate cfDNA fragmentation. qPCR reactions were performed in triplicate using a CFX96 Touch Real-Time PCR Detection System (Bio-Rad Laboratories, Hercules, USA) as described previously (79). No Template Control (NTC) was used for every assay. Analysis was done using CFX Maestro Software (Bio-Rad Laboratories, Hercules, USA). Absolute quantification of DNA in each sample was determined by a standard curve with serial dilutions of human male genomic DNA (Promega, Madison, WI, USA) and expressed as ng of cfDNA per 1 mL of blood plasma.

### Statistical Analysis

Statistical analysis was performed using Minitab statistical software, version 19.1.1 (Minitab, LLC, 2021., Centennial, USA). The level of significance was set at *P* < 0.05. Mixed effect regression model was used to perform analysis, where change in individual measurement was set as dependent variable. Time point (weeks) as well as sampling points (pre-dive or post-dive) were set as independent variables, with sampling point variable being nested inside the time point variable. As a random intercept, subjects IDs were used to minimize effects of interindividual variability. For pairwise comparisons, Tukey *post hoc* test was used. To account for multiple testing, Benjamini-Hochberg correction was used to adjust the *P*-values. All data were presented with corresponding center value and scatter measures based on results of normality testing with Kolmogorov-Smirnov test. Before statistical analysis of N-glycans, 9 derived traits were calculated for IgG N-glycans from 24 directly measured glycan traits, while for human plasma protein N-glycans, 16 derived traits were calculated using 39 directly measured glycan traits and calculation formulas from Table 1.

## Results

To investigate the effect of five consecutive rSCUBA dives on CV system and underlying immunological background, we conducted a longitudinal intervention study and measured concentrations of specific biomarkers in six time points: immediately before and after the first, third and fifth dives. The diver’s anthropometric data, expressed as median and IQR, were: height 1.80 (1.76–1.86) m, weight 85 (77–93) kg, and body mass index (BMI) 26.4 (23.5–28.5) kg/m^2^, respectively.

### Copeptin

**FIGURE 2:**
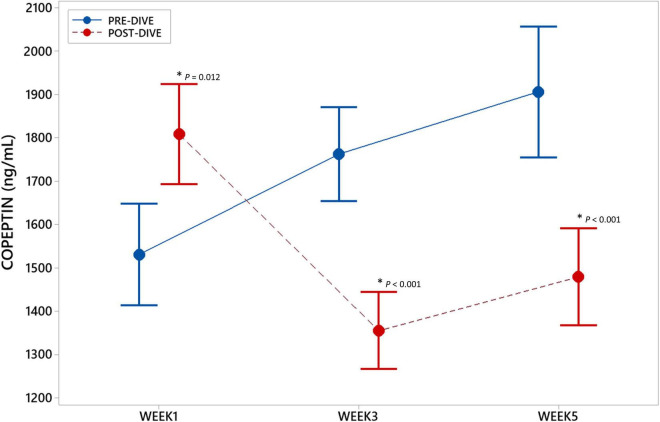
Interval plot (mean ± SE) showing changes in copeptin (CPP) serum concentrations throughout all sampling time points. *Statistically significant as compared to the corresponding pre-dive value.

To examine the effect of repeated rSCUBA diving on underlying CVS molecular processes, copeptin (CPP) concentrations in serum samples were measured. CPP concentration increased statistically significant (*P* = 0.012) after the first dive as compared to the baseline value. However, third and fifth dive caused statistically significant decrease in CPP concentration (W3: *P* < 0.001; W5: *P* < 0.001) (Figure 2). Significant effect of five consecutive dives on CPP concentration throughout all 5 weeks was not observed (*P* = 0.175).

### Differential Blood Count Parameters and Neutrophil-to-Lymphocyte Ratio

**FIGURE 3:**
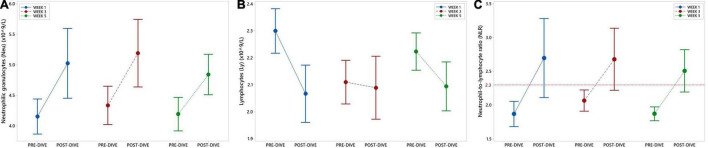
Interval plots (mean ± SE) showing changes in absolute number of neutrophilic granulocytes (Neu) **(A)** and lymphocytes (Ly) **(B)** absolute numbers, as well as neutrophil-to-lymphocyte ratio (NLR) **(C)** across all sampling time points.

Absolute number of neutrophilic granulocytes (Neu) increased statistically significant after the first and third dives compared to the corresponding pre-dive values (W1: *P* = 0.031; W3: *P* = 0.034) (Figure 3A and Table 2). Lymphocyte absolute number (Ly) decreased significantly only in week 1 (W1: *P* = 0.049) (Figure 3B and Table 2). Calculated neutrophil-to-lymphocyte ratio (NLR) increased statistically significant, also only after the first dive (W1: *P* = 0.021) (Figure 3C and Table 2). An effect of five consecutive dives on the differential blood count (DBC) parameters was not observed (Neu: *P* = 0.667; Ly: *P* = 0.580; NLR: *P* = 0.761) (Table 3).

**TABLE 2:** Estimated effects of every single dive on levels of specific laboratory parameters, and their respective adjusted *P*-values.

	Post-dive vs. Pre-dive (Week 1)		Post-dive vs. Pre-dive (Week 3)		Post-dive vs. Pre-dive (Week 5)	
Parameter	Estimated effect coefficient	Adj. *P*-value	Estimated effect coefficient	Adj. *P*-value	Estimated effect coefficient	Adj. *P*-value
Neu	**0.4364**	↗ **0.031**	**0.4286**	↗ **0.034**	0.3246	0.106
Ly	**−0.1164**	↘ **0.049**	−0.0104	0.859	−0.0646	0.270
NLR	**0.4143**	↗ **0.021**	0.3054	0.087	0.3176	0.075
IgA	**0.0321**	↗ **0.023**	**0.0354**	↗ **0.013**	**0.0396**	↗ **0.006**
IgG	**0.2075**	↗ **0.001**	**0.1893**	↗ **0.003**	**0.2654**	↗ **0.001**
IgM	**0.0137**	↗ **0.023**	**0.0167**	↗ **0.006**	**0.0177**	↗ **0.004**
C3	0.0146	0.144	0.0192	0.056	**0.0212**	↗**0.035**
C4	0.0038	0.301	0.0038	0.301	0.0043	0.246

*Neu – absolute number of neutrophilic granulocytes, Ly – absolute number of lymphocytes, NLR – neutrophil-to-lymphocyte ratio, IgA – immunoglobulin A, IgG – immunoglobulin G, IgM – immunoglobulin M, TRF – transferrin, C3 – C3 complement component, C4 – C4 complement component, ↗ (green) statistically significant increase (P < 0.05), ↘ (red) statistically significant decrease (P < 0.05), bold effect coefficients are statistically significant.*

**TABLE 3:** Estimated effects of five consecutive dives on levels of specific laboratory parameters, and their respective adjusted *P*-values.

	Week 3 vs. Week 1		Week 5 vs. Week 1		Week 5 vs. Week 3	
Parameter	Estimated effect coefficient	Adj. *P*-value	Estimated effect coefficient	Adj. *P*-value	Estimated effect coefficient	Adj. *P*-value
Neu	0.1740	0.809	−0.0720	0.965	−0.2460	0.656
Ly	−0.0839	0.567	−0.0246	0.952	0.0593	0.752
NLR	0.0910	0.930	−0.0930	0.925	−0.1840	0.740
IgA	0.0168	0.668	**0.0532**	↗ **0.022**	0.0364	0.158
IgG	0.1796	0.097	**0.2107**	↗ **0.042**	0.0311	0.930
IgM	0.0136	0.236	**0.0226**	↗ **0.022**	0.0089	0.529
C3	−0.0216	0.277	0.0108	0.723	0.0323	0.061
C4	−0.0096	0.157	0.0052	0.574	**0.0148**	↗ **0.015**

*Neu – absolute number of neutrophilic granulocytes, Ly – absolute number of lymphocytes, NLR – neutrophil-to-lymphocyte ratio, IgA – immunoglobulin A, IgG – immunoglobulin G, IgM – immunoglobulin M, TRF – transferrin, C3 – C3 complement component, C4 – C4 complement component, ↗ (green) statistically significant increase (P < 0.05), bold effect coefficients are statistically significant.*

### Immunoglobulins

**FIGURE 4:**
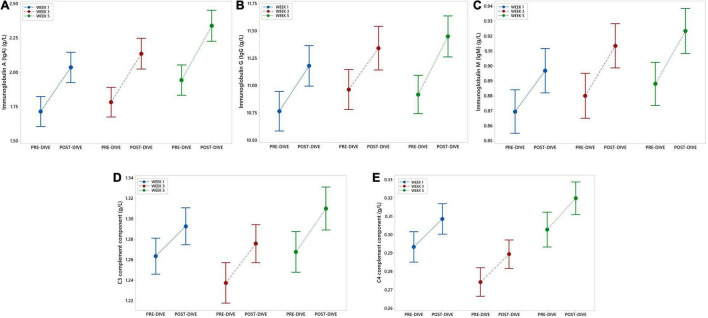
Interval plots (mean ± SE) showing changes in immunoglobulin (Ig) IgA **(A)**, IgG **(B)**, and IgM **(C)**, as well as C3 **(D)** and C4 **(E)** complement components serum concentrations across all sampling time points.

IgA, IgG, and IgM serum post-dive concentrations increased statistically significant as compared to the corresponding pre-dive values (Figures 4A–C and Table 2). Calculated effect of five consecutive dives showed significant increases in levels of all measured immunoglobulins in the last week (W5) compared to the week 1 (W1) values (IgA: *P* = 0.022; IgG: *P* = 0.042; IgM: *P* = 0.022) (Figures 4A–C and Table 3). All measured values remained in the corresponding reference ranges.

### Complement Components C3 and C4

No statistically significant changes in concentration of C3 and C4 complement components after single dives were found, except that slight increase in C3 level after the fifth dive compared to the corresponding pre-dive value was observed (*P* = 0.035) (Figure 4D and Table 2). Statistically significant effects of repeated dives on C3 concentration throughout all 5 weeks were also not observed (C3: *P* = 0.070), whereas for C4 concentration a slight increase was observed in week 5 (W5) compared to week 3 (W3) (*P* = 0.015) (Figure 4E and Table 3), but mainly due to the decrease in week 3 (W3) compared to week 1 (W1). All measured values remained in the corresponding reference ranges.

### N-Glycans

Effect of the five consecutive dives on the IgG and TPP derived N-glycan traits was estimated only as a trend of change within all 5 weeks of the study. Changes between pre-dive and post-dive measurements were not the subject of the analyses since nature of changes in glycan trends were expected to appear only during the studied period. More precisely, the effects of repeated rSCUBA diving on N-glycans were observed as changes occurred in the third and fifth week compared to the values determined in the first week.

#### IgG N-Glycosylation

**FIGURE 5:**
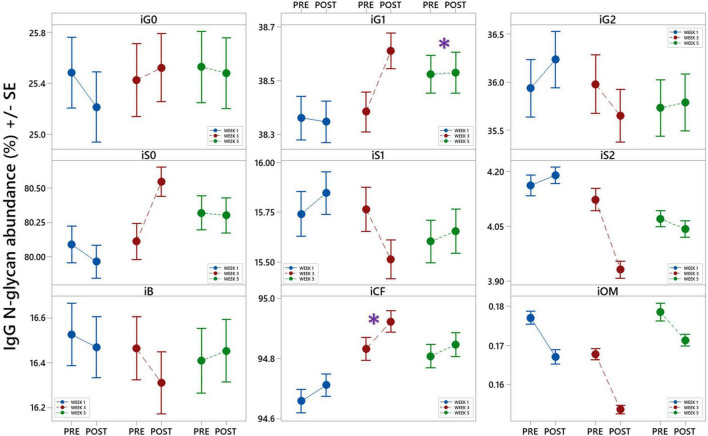
Interval plots (mean ± SE) showing changes in 9 different derived IgG N-glycan traits across all sampling time points. *Statistically significant as compared to week 1 (W1). i – refers to IgG glycans, G0 – agalactosylation, G1 – monogalactosylation, G2 – digalactosylation, S0 – neutral glycans, S1 – monosialylation, S2 – disialylation, B – bisecting glycans, CF – core-fucosylation, OM – oligomannose glycans.

The calculated effect of repeated rSCUBA diving on IgG N-glycans showed statistically significant changes in the third and fifth week compared to the first week, in levels of monogalactosylated N-glycans (iG1), which increased in the fifth week (W5 vs. W1, *P* = 0.041), and core-fucosylated N-glycans (iCF), which also increased in the third week (W3 vs. W1, *P* = 0.013) (Table 4). Figure 5 shows changes of each IgG glycosylation derived trait from baseline to the end of the study represented by mean ± SE interval plots.

**TABLE 4:** Estimated effects of five consecutive dives on levels of individual derived IgG and total plasma protein (TPP) N-glycan traits, and their respective adjusted *P*-values.

	Week 3 vs. Week 1		Week 5 vs. Week 1		Week 5 vs. Week 3	
	Estimated effect	Adj. *P*-value	Estimated effect	Adj. *P*-value	Estimated effect	Adj. *P*-value
	coefficient		coefficient		coefficient	
**Derived IgG N-glycan trait**						
iG0	0.1260	0.474	0.1570	0.320	0.0300	0.957
iG1	0.1432	0.105	**0.1725**	↗ **0.041**	0.0293	0.907
iG2	–0.2720	0.168	–0.3250	0.081	–0.0530	0.932
iS0	0.3010	0.078	0.2820	0.105	–0.0190	0.989
iS1	–0.1546	0.155	–0.1646	0.122	–0.0100	0.992
iS2	–0.1479	0.063	–0.1186	0.164	0.0293	0.892
iB	–0.1111	0.055	–0.0664	0.342	0.0446	0.613
iCF	**0.1932**	↗ **0.013**	0.1429	0.086	–0.0504	0.728
iOM	–0.0114	0.269	0.0029	0.919	0.0143	0.132
**Derived TPP N-glycan trait**						
LB	0.194	0.769	–0.269	0.605	–0.463	0.232
HB	–0.203	0.750	0.263	0.619	0.466	0.228
G0	**−0.779**	↘ **0.004**	**−0.635**	↘ **0.024**	0.145	0.812
G1	**−0.980**	↘ **0.047**	–0.927	0.064	0.054	0.990
G2	**1.883**	↗ **0.013**	1.211	0.152	–0.671	0.553
G3	–0.026	0.993	0.381	0.231	0.407	0.190
G4	–0.178	0.141	–0.119	0.410	0.059	0.800
S0	**−2.010**	↘ **0.025**	–1.755	0.057	0.256	0.938
S1	0.202	0.680	0.273	0.496	0.071	0.953
S2	**1.993**	↗ **0.008**	1.427	0.077	–0.566	0.657
S3	–0.046	0.966	0.155	0.673	0.201	0.517
S4	–0.148	0.123	–0.107	0.326	0.041	0.848
CF	**−2.022**	↘ **0.042**	**−2.034**	↘ **0.040**	–0.013	1.000
AF	–0.134	0.634	–0.009	0.998	0.125	0.672

*i – IgG glycans, G0 – agalactosylation, G1 – monogalactosylation, G2 – digalactosylation, G3 – trigalactosylation, G4 – tetragalactosylation, S0 – neutral glycans, S1 – monosialylation, S2 – disialylation, S3 – trisialylation, S4 – tetrasialylation, B – bisecting glycans, CF – core-fucosylation, OM – oligomannose glycans, LB – low branching glycans, HB – high branching glycans, AF – antennary fucosylation. ↗ (green) statistically significant increase (P < 0.05), ↘ (red) statistically significant decrease (P < 0.05), bold effect coefficients are statistically significant.*

#### Total Plasma Proteins N-Glycosylation

**FIGURE 6:**
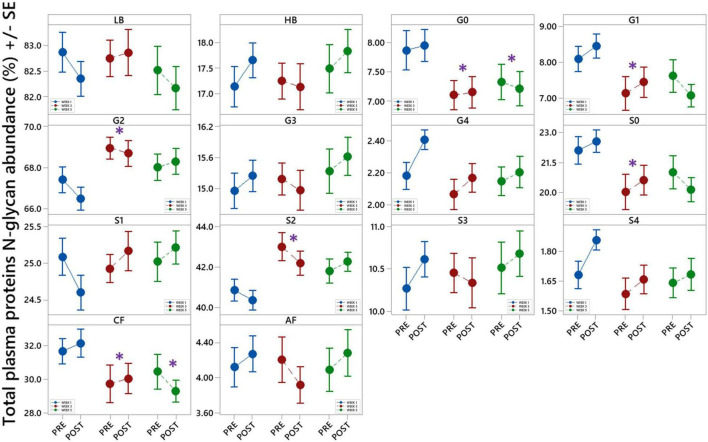
Interval plots (mean ± SE) showing changes in 14 different derived total plasma proteins (TPP) N-glycan traits across all sampling time points. *Statistically significant as compared to week 1 (W1). LB – low branching glycans, HB – high branching glycans, G0 – agalactosylation, G1 – monogalactosylation, G2 – digalactosylation, G3 – trigalactosylation, G4 – tetragalactosylation, S0 – neutral glycans, S1 – monosialylation, S2 – disialylation, S3 – trisialylation, S4 – tetrasialylation, CF – core fucosylation, AF - antennary fucosylation.

Changes in total plasma protein (TPP) N-glycosylation are shown in Figure 6 and Table 4. Significant decreases in agalactosylated (G0) and core-fucosylated (CF) N-glycans were found over the studied period (W3 vs. W1 and W5 vs. W1). Further, increase in digalactosylated (G2) and disialylated (S2) N-glycans as well as decreased monogalactosylated (G1) and asialylated (S0) were observed in week 3 (W3 vs. W1).

### Cell-Free DNA and Cell-Free DNA Integrity Index

**FIGURE 7:**
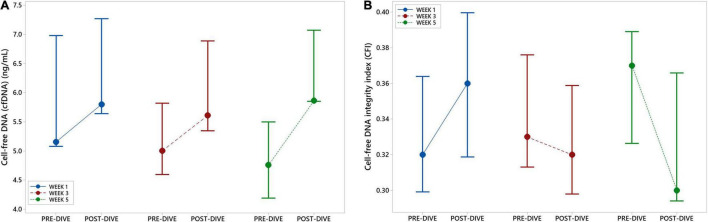
Interval plots (median ± SE) showing changes in cell-free DNA (cfDNA) plasma concentration **(A)** and cell-free DNA integrity index (CFI) **(B)** across all sampling time points.

Positive and negative variations in cfDNA concentration before and after each dive were observed, with no general trends detectable, except in the fifth dive when a statistically significant increase was detected (*P* = 0.037) (Figure 7A and Table 5). Yet, this increase did not exceed 25% of the pre-dive median value. The concentration of cfDNA between weeks did not alter in response to rSCUBA diving (*P* = 0.458). Regarding CFI, there was no observed difference between weeks (*P* = 0.822), as well as before and after each dive (*P* = 0.277). Medians of CFI were relatively stable over the study period varying from 0.30 to 0.37 (Figure 7B and Table 5).

**TABLE 5:** Estimated effects of every single dive on the concentration [median (interquartile range)] of cell-free DNA (cfDNA) and cfDNA integrity index (CFI), and their respective and adjusted *P*-values.

cfDNA (ng/mL)	Median (interquartile range)		Estimated effect coefficient	Adj. *P*-value
	Pre-dive	Post-dive		
Week 1	5.15 (2.95–9.45)	5.79 (4.23–9.46)	0.2122	0.578
Week 3	5.00 (2.93–6.84)	5.61 (3.97–8.05)	0.4546	0.236
Week 5	4.76 (2.41–6.37)	5.86 (4.65–7.58)	**0.8087**	↗**0.037**

**CFI (cfDNA LINE-1 82-bp/224-bp)**	**Median (interquartile range)**		**Estimated effect coefficient**	**Adj. *P*-value**
	**Pre-dive**	**Post-dive**		

Week 1	0.32 (0.30–0.38)	0.35 (0.32–0.37)	0.0138	0.199
Week 3	0.33 (0.31–0.37)	0.32 (0.31–0.35)	−0.0081	0.451
Week 5	0.37 (0.31–0.40)	0.30 (0.29–0.37)	−0.0138	0.199

*↗ (green) statistically significant increase (P < 0.05), bold effect coefficients are statistically significant.*

## Discussion

In this study we present the results of a longitudinal intervention study, in which fourteen divers who performed five dives, one per week, on the depth 20–30 m that lasted 30 min, after a non-dive period of 5 months have been included. We measured cardiac and inflammatory markers in samples obtained in six time points: before and after the first, third, and fifth dives.

### Copeptin

The environmental (e.g., high pressure, immersion and cold), as well as emotional stressors that follow diving may induce hemodynamic and volemic changes *via* the AVP (80). In this study, changes in CPP serum concentration showed interesting dynamics; namely, the first dive was followed by an increase, while the third and fifth dives by a decrease in CPP level, but no cumulative effect was noticed. The regulation of AVP release is complex and affected by numerous factors, organic and psychological. The factors that induce AVP release, in addition to hyperosmolarity, are hypoxia (10) that occurs during rSCUBA diving (11) due to peripheral vasoconstriction triggered by exposure to coldness during the dive (10), as well as IL-6 (17) released by active muscles (81) which was found to be increased after every rSCUBA dive (4). Many studies have shown up-regulation of CPP level during exercise in different settings (82–85), although regulatory mechanisms of AVP release triggered by exercise are not fully understood. Namely, increased osmolality and decreased plasma volume, potent inducers of AVP release, cannot fully explain observed increases in CPP level in exercising participants. The study in which possible involvement of IL-1β in the control of AVP release was investigated showed that exercise induces plasma copeptin levels was independent of sodium levels or fluid loss and not regulated by the IL-1 pathway (82). In opposite, water immersion is followed by AVP suppression by mechanisms that are incompletely defined, but it appears that not only cardiopulmonary mechanoreceptors but also arterial baroreceptors mediate the response (86). It was shown that the exposure of 3 atm air (that corresponds to the dive of 20 m depth) for 1 hour leads to a significant reduction of plasma osmolality and the reduction in AVP plasma level (87). Thus, the AVP release during the dive is a result of combined actions of stimulating and inhibitory factors. It is worth to mention that in the case of the continuous action of stimulating factors, prolonged secretion of arginine vasopressin (AVP), *via* V1A receptors expressed on cardiac tissue, stimulates protein synthesis in myocytes and development of myocardial hypertrophy, decreases heart contractility, activates cardiac fibroblasts, thus promoting myocardial fibrosis (19). It seems that during the first dive the inhibitory factors were overridden by stimulating factors and resulted in increased CPP level, whereas repeated rSCUBA diving promoted activity of inhibitory factors thus resulting in decrease in CPP levels after the third and fifth dives. These results can be related to the fact that the first dive was performed after non-diving period of 5 months, and obviously triggered intensive neurohormonal stress response, which was alleviated by regular repletion of diving. Since repeated dives were followed by decreases in CPP level the possibility of any further cardiac damage may be excluded. This conclusion is additionally supported by our previous findings of continuous decrease in hs-TnI concentration during the observed period (4). Further, the dynamics of CPP changes advocate our previous presumption that increase in NT-proBNP concentration observed over the studied period was not associated with any cardiac damage or pathological remodeling but only with positive and protective role for cardiac myocytes (4).

Our previous study on the effects of s rSCUBA dive of the same profile (30 m during 30 min) on the oxidant/antioxidant status, and expression of NAD^+^-dependent histone deacetylase, sirtuin 1 (SIRT1) and sirtuin 3 (SIRT3) suggested that this diving profile represents a special form of hormesis in which low-to-moderate increase of ROS may have beneficial effects on health (88). Particularly interesting results were decreased *SIRT1* gene expression immediately and 3 hours after diving and increased *SIRT3* gene expression 6 hours after the dive in peripheral blood mononuclear cells. Both enzymes are involved in deacetylation of forkhead Box O3a (FOXO3a), a transcriptional factor that induces expression of antioxidants, superoxide dismutase 2 (SOD2; Mn-SOD) and catalase (CAT) (89, 90), thus exerting cardioprotective effects. Yet, SIRT1’s cardioprotective effect was observed only at low dosage whereas overexpression of SIRT1 in mouse hearts was shown to produce hypertrophic cardiomyopathy (91). SIRT3 also exerts its cardioprotective effect through the preservation of the ATP biosynthetic capacity of the heart (92). It is the main mitochondrial sirtuin that among various substrates deacetylates and activates LKB1, a serine/threonine kinase that phosphorylates and activates adenosine monophosphate (AMP)-activated protein kinase (AMPK) (93). AMPK is a cellular energy sensor that is activated by cellular stresses that deplete ATP and elevate AMP, such as glucose deprivation, hypoxia, ischemia, oxidative stress, and hyperosmotic stress (94), but also by exposure to coldness (95) and exercise (96, 97). It has been recently described as a key player in metabolic adaptations to endurance training (98). In the heart, AMPK activation leads to increased mitochondrial biogenesis through increased expression of peroxisome proliferator-activated receptor gamma coactivator 1-alpha (PGC1α) (99). By promoting autophagy (100) and suppressing fibrosis (101), AMPK contributes cardioprotection. It was shown that exogenous NAD blocks cardiac hypertrophic response *via* activation of the SIRT3-LKB1-AMPK pathway (93). Although in this study we have no possibility to measure either SIRT3 or activated AMPK (pAMPK), based on our previous results (88) here we presume that SCUBA diving due to the environmental conditions (oxidative stress, coldness, muscle activation, peripheral hypoxia) is followed by low NAD^+^ content in the cardiomyocytes, which in turn results in up-regulation of SIRT3 that leads to activation of AMPK, thus promoting cardioprotective effects of SCUBA diving.

### Immune Response

A continuous moderate physical exercise has multiple beneficial effects on overall health and mental well-being and reduces the risk of various chronic diseases, including infections and inflammatory diseases. Still, an acute episode of physical activity activates various components of immune system, that in healthy individuals results in transient inflammatory response that includes increases in different cytokines. The main source of cytokines during the physical exercise are active muscles. They secrete several interleukins (ILs), IL-4, IL-6, IL-7, IL-8, and IL-15, which are part of the muscle secretome that consists of several hundred secreted peptides, called myokines (81). Although IL-6 has both proinflammatory and anti-inflammatory properties, in the context of exercise, IL-6 exerts a strong anti-inflammatory effect by inducing the release of anti-inflammatory cytokines, specifically IL-1 receptor antagonist (IL-1RA) and IL-10, from blood mononuclear cells (102), but also by decreasing circulating levels of tumor necrosis factor alpha (TNFα) (103). By increasing lipolysis and fat oxidation in adipose tissue IL-6 also induces other healthy metabolic effects that, in turn, decrease the risk of CV diseases (104). As previously mentioned, we have recently shown that rSCUBA diving induces IL-6 production after every dive, and that this effect becomes more pronounced after five dives, suggesting its anti-inflammatory effect (4). In the recent study in which effect of air and trimix on the inflammatory response following dives to 50 meters of sea water was studied, IL-6 values showed a slight increase, while IL-8 decreased in both groups, without significant variation between the groups (105). In the study, in which diving environmental conditions were simulated in hyperbaric chambers (18 m of sea water (msw) for 60 min or 30 msw for 35 min), increases in IL-1β during compression and 2 hours post-decompression were observed (106).

#### Differential Blood Count Parameters and Neutrophil-to-Lymphocyte Ratio

Slight increases in neutrophil and lymphocyte counts were observed after all three dives, but statistically significant changes in neutrophil count were observed after the first and third dives whereas decreases in lymphocyte count only after the first dive. Similar changes were observed in our previous study, in which the increase in neutrophil count, observed immediately after the dive, continued during the recovery period (up to 6 hours after the dive) (107). In that study we noticed that the slight decline in lymphocyte count observed immediately after the dive, were growing during the recovery period, and after 6 hours it was statistically higher compared to the pre-dive values. Similar dynamics was also noticed in the total leukocyte count. The increases of two- to threefold in leukocyte count even after short dynamic exercise and up to fivefold after prolonged endurance exercise have been reported (108). Yet, it this study changes in neutrophil and lymphocyte counts were slight and remained within 25% of the pre-dive values. It is well-known that the exercise-induced leukocytosis is a transient phenomenon, with normal leukocyte and leukocyte subtype distribution typically returning to pre-exercise levels within 6–24 h after exercise cessation (109), so we presume that similar dynamics occurs also after the rSCUBA diving. It seems that immediately after the physical activity and during the early recovery stages (within 30–60 min of exercise cessation), a rapid decrease in the lymphocyte count occurs in parallel with an increase in neutrophil count. In general, exercise-induced increase in cardiac output and blood flow, causes a transient interchange of leukocytes and other immune components, e.g., immunoglobulins, anti-inflammatory cytokines, complement, between blood and tissues and over time has a cumulative effect that enhances immunosurveillance against various exogenous and endogenous harmful agents, thus decreasing systemic inflammation and contributing to the general health (110). It is important to emphasize that these positive effects have been observed mainly after physical activity of moderate intensity, whereas numerous studies suggested that prolonged and high exercise training workloads followed by physiological, metabolic, and psychological stress lead to immune dysfunction, inflammation, oxidative stress, and muscle damage and in general have negative impact on health (111, 112).

The cut-off values of neutrophil-to-lymphocyte ratio (NLR) to measure the intensity of inflammation were recently refined in line with clinical trials and observations (113). A normal NLR is about 1–3 (114), although the values between 2.3 and 3 should be considered as a “gray zone” that reflects latent, subclinical, or low-grade inflammation/stress (NLR 2.3–3.0) (27). In our study all pre-dive NLR values were below 2.3, whereas all post-dive values were above 2.3 (W1, W3: 2.7; W5: 2.5). Although every dive triggered a slight increase in NLR, statistically significant rise was observed only after the first dives. Concomitant increase in NLR and decrease in copeptin level, together with our previous findings of most pronounced changes in hs-TnI and CK-MB (4) after the first dive suggest that it caused more pronounced disturbance in cardiac tissue compared to two other dives. This is not surprising since the first dive was performed after a non-dive period of 5 months and obviously triggered more intensive stress response and inflammation compared to the next two observed dives, suggesting an adaptation of organism to the repeatedly performed rSCUBA diving. To the best of our knowledge, this is the first time that NLR has been considered in relation to rSCUBA diving. A recent study on animal model of decompression sickness (DCS) showed that the NRL was significantly higher in DCS-resistant individuals compared with non-resistant individuals, but the dilemma whether lower-grade systemic inflammation was already present in DCS-resistant individuals or whether the resistance to DCS was associated with an increased immune capacity that would enhance an acute proinflammatory response to diving and/or decompression-related stress remained unanswered (115). In brief, the first dive after a long non-dive period caused disturbances in cardiac tissue that were followed by inflammatory response, which may have contributed to the repair of cardiac microdamages. Yet, since these changes were mild and have not been observed in the next dives, one could conclude that regular repetition of rSCUBA diving leads to the adaptation of cardiovascular and immune system to this physical exertion in extreme environmental conditions.

#### Immunoglobulins

Increases in post-dive levels of IgA, IgG, and IgM compared to the corresponding pre-dive levels in all three studied points suggest that single rSCUBA dive triggers release of Igs from immune niches, since that period is too short for *de novo* synthesis. Interestingly, Karacabey and coworkers observed the increase in IgA, IgG, and IgM levels only 2 days after aerobic exercise performed by elite athletes, so it seems that aerobic exercise triggered *de novo* synthesis of Igs (29, 30). Furthermore, repetition of rSCUBA diving led to the increases in levels of all measured Ig classes (IgA, IgG, and IgM), during the whole studied period of 1 month, with statistically significant differences observed between W1 and W5, with remark that all measured values remained in corresponding reference range. These data suggest that rSCUBA diving is followed by increases in IgA, IgG, and IgM levels, thus advocating its favorable impact on the immune system and cardioprotective effect.

#### Complement Components C3 and C4

No statistically significant changes in complement components C3 and C4 levels were found after single rSCUBA dive and after 5 repeated dives (W5 vs. W1). The only statistically significant changes observed were slight increases in C3 level after the first dive (post- vs. pre-dive values) and in C4 level in week 5 (W5) compared to week 3 (W3), with a note that C4 level deceased, although not significantly, in week 3 (W3) compared to week 1 (W1). Published scientific data on the effects of exercise on C3 and C4 levels are not consistent because it was shown that after physical exercise levels of C3 and C4 decline (29–31, 45), remain unchanged (42, 44) or increase (43). Yet, many findings corroborate that increases in C3 and C4 negatively corelate with health. Namely, global physical fitness was found to be inversely associated with C3 and C4 levels in children, as well as C4 in adolescent (47). Further, levels C3 and C4 levels were found to be lower in sportsmen compared to sedentary (46). Even in the study in which increased levels of C3 and C4 during and after graded maximal treadmill test (compared to basal levels) in both marathon runners and sedentary controls were found, the levels in athletes were significantly lower compared to the controls (43). It should be emphasized that in our study all values remained within corresponding reference range, so changes in C3 and C4 levels triggered by rSCUBA diving are the most probably consequence of transient interchange of complement components between blood and tissues. Furthermore, it was shown that decreased levels of C3 and C4 noticed immediately after physical exercise returned to the pre-exercise levels within 4 hours (29, 30), so additional research on dynamics of changes in C3 and C4 levels after rSCUBA diving during the recovery period is recommended. Taken together, these findings suggest that repeated rSCUBA diving does not have any significant impact on complement system activation.

### N-Glycans

#### IgG N-Glycosylation

In this study we also investigated the effects of repeated rSCUBA diving on IgG and TPP N-glycosylation profiles that reflect inflammation status of the human body. In case of IgG N-glycosylation profile, among nine measured glycosylation traits, two of them showed statistically significant changes. The observed increase in monogalyctosylated N-glycans after five dives (W5 compared to W1) is related to suppressed complement activation by IgG, which consequently decreases its inflammatory potential (51, 52). In parallel, increase in core-fucosylated N-glycans, observed in week 3 (W3 vs. W1), significantly reduces IgG affinity for FcγRIIIA receptors and activation of ADCC (49, 50). Therefore, the observed changes suggest that repeated rSCUBA diving promote anti-inflammatory effects.

#### Total Plasma Proteins N-Glycosylation

To explore whether repeated rSCUBA diving results in more general change in glycosylation of multiple proteins, we also investigated TPP glycosylation. Decreases in total agalactosylated N-glycans found over the studied period (W3 vs. W1 and W5 vs. W1), corroborate anti-inflammatory effects of repeated rSCUBA diving (116, 117). This finding was supported by increases in digalactosylated and asialylated (neutral) N-glycans in week 3 (W3 vs. W1), which are also glycan trails positively correlated with anti-inflammatory effects. Interestingly, opposite to the finding of increase in IgG core-fucosylated N-glycans, the decreases in TPP core-fucosylated N-glycans over studied period (W3 vs. W1, and W5 vs. W1) as well as increase in disialylated N-glycans in week 3 (W3 vs. W1) were observed. Yet, due to the relative quantification of a particular glycan trait, in relation to all N-glycans released from all plasma proteins, it is apparent that observed changes are the consequence of the changed ratio of the numerous plasma glycproteins as well as their glycosylation. Due to the high portion of IgG in total plasma proteins (10–20%), IgG N-glycans represent significant part of plasma N-glycans, so changes in IgG N-glycosylation are highly reflected in plasma N-glycome. Since changes in IgG N-glycans after repeated SCUBA diving are not reflected in changes of plasma N-glycome, it seems that this physical activity affects N-glycosylation of other plasma glycoproteins, such as transferrin, IgA, alpha-1-acid glycoprotein, haptoglobin as well as C3 and C4 more significantly, thus resulting in observed changes.

To sum up, observed N-glycosylation changes caused by repeated rSCUBA diving, associated with lower inflammatory status and younger age (117, 118) corroborate positive effects of repeated rSCUBA diving on general health. Yet, it would be interesting to explore N-glycosylation of other plasma glycoproteins, which would give better insight in the effects of repeated rSCUBA diving on N-glycosylation, but also to investigate for how long these changes remain detectable after cessation of diving period.

The obtained results related to the immune response suggest that repeated rSCUBA diving promote anti-inflammatory status in the organism. Still, with a purpose to enhance positive effects of exercise, many athletes, including SCUBA divers use various nutraceuticals, including antioxidants, vitamins, minerals, dietary supplements, as well as different herbal remedies. Among the latest, polyphenols, especially flavonoids found in plant-based foods, attract a lot of scientific, but also clinical attention due to their proven anti-inflammatory (119), anti-cancerogenic (120), antiviral (121), anti-oxidative and cardioprotective (122) properties. Many of them affect production of different components of immune system thus modulating numerous immune reactions, but also other physiological processes, through various signaling pathways (123, 124). For instance, quercetin showed inhibitory effect on production of complement components (125, 126), interleukins (127, 128), and immunoglobulins (125, 129). However, these effects should be considered with special attention when the use of flavonoids by athletes is reviewed. For example, inhibition of IL-6 production by quercetin (127) may suppress AVP production thus preventing an adequate retention of water after its loss due to increased sweating during the exercise, but also possibly abolish IL-6 anti-inflammatory effects and lead to pro-inflammatory response due to diminished production of IL-1RA and IL-10 (102) and increased production of TNFα (103). Yet, it was shown that quercetin mitigate postexercise increases in IL-8 and TNFα plasma levels and diminished expression of leukocyte IL-8 and IL-10 mRNA in trained male cyclists (130). Although inhibitory effects of flavonoids in general promote anti-inflammatory status of the organism, they should be used by athletes with cautious because acute inflammation triggered by physical exercise is a part of physiological response of organism directed to protection and most probably to the successful recovery after physical load. Therefore, prevention or suppression of acute inflammatory response after physical exercise by flavonoids or any other nutraceuticals may lead to undesirable or even adverse effects. However, generally accepted beneficial effects of nutraceuticals, such as enhancing endurance exercise capacity (131), contributing antioxidative status (132), preventing exercise-induced muscle damage (133), although not always scientifically proven, lead to massive and widespread self-prescribed use of flavonoids by athletes. Therefore, comprehensive studies of the effects of nutraceuticals on physiological processes triggered by physical exercise at the molecular levels are of utmost importance and need.

### Cell-Free DNA and Cell-Free DNA Integrity Index

Different types of vigorous, exhaustive, endurance, and strength exercise cause significant elevation of plasma cfDNA concentration, reaching ten-folds increase after a single bout of exercise (63, 65, 67, 134). Yet, all increases observed in this study were very mild (up to 25% of the pre-dive values), whereas the only statistically significant difference was recorded after the fifth dive. An increase in cfDNA level after intensive exercise is strongly correlated with other markers of muscle damage, e.g., creatine kinase (CK) and lactate (133), so it was mainly attributed to muscular damage (64, 70). The present results are in accordance with our previously findings that rSCUBA diving triggers very mild myocyte damage, since only slight and transient increases in myoglobin, galectin-3, CK, and LDH levels were detected after the dives (4). Exercise-induced increase in cfDNA level could be also a consequence of enhanced leukocytes activity and apoptosis (64), or formation of intravascular neutrophil extracellular traps (NETs) (71). As previously shown, rSCUBA diving is not accompanied by changes in leukocyte count (4), whereas there is no evidence that slight increases in neutrophil count (observed after the first and third dives, but not after the fifth dive when statistically significant increase in cfDNA level was noticed), comprise their activation and formation of neutrophil extracellular traps. Cellular necrosis occurring due to high-intensive strength training, because of mechanical, energetic, and/or ischemic stress is also a source of release of cfDNA in circulation (66). Although rSCUBA diving is followed by peripheral vasoconstriction due to hyperbaric conditions and coldness, and may cause hypoxia, our results on cfDNA support assumption that it does not cause cellular necrosis. In addition, cfDNA release has also been associated with heart failure and myocardial infarction because of ischemia and cardiomyocyte death (135, 136), but these events can be also excluded, not only because of findings in cfDNA level, but mostly on our recent results showing only physiological changes in cardiovascular system after rSCUBA diving (4). Absence of cellular necrosis after rSCUBA diving was also confirmed by cfDNA integrity index (CFI) values, since no statistically significant changes in CFI in any studied time point were observed. Namely, higher CFI implies necrotic processes because necrotic cells usually excrete longer cfDNA fragments, while shorter ones are being released *via* apoptosis (72). To conclude, observed changes in cfDNA level and CFI support the assumption that rSCUBA diving causes neither exercise-induced inflammation nor tissue injury.

### Study Limitations

To the best of our knowledge and based on current available scientific literature analysis, this is the first study to evaluate the effect of five repeated rSCUBA dives on specific cardiac (copeptin) and inflammatory (Igs, C3, C4, Neu, Ly, NLR, N-glycosylation of IgG and TPP) markers as well as cfDNA and its integrity. However, it has several limitations that must be emphasized.

The study did not include female participants, so we were not able to investigate the impact of gender on obtained results. Furthermore, since this study was conducted over a relatively short period of time (1 month), a longer study conducted under the same diving conditions or additional sampling after the final dive could reveal more information on the cumulative effect of rSCUBA diving on the blood concentration of the selected biomarkers. Further, it was impossible to compare the obtained results with previous studies because comparable ones have not been conducted so far. Finally, although this was one of the studies with the largest number of participants among all studies conducted so far on recreational SCUBA divers, a small number of divers made it impossible to group them according to age and diving experience. Therefore, even though we were aware of the possible impact, it was impossible to fully take these variables into the interpretation of the obtained results.

## Conclusion

Recreational SCUBA diving practiced on a regular basis induce cardiac adaptive response and promotes anti-inflammatory status of the organism thus conferring cardioprotection as well as multiple health benefits. Yet, the impact of exercise on the organism is highly dependent on the frequency and intensity of exercise, the duration and load of training, and in extreme sports, such as diving, on environmental conditions. Therefore, it is of utmost importance to intensify scientific research on the effects of different types of diving on physiological processes at molecular level thus contributing to the elucidation of impact of this physical activity in extreme environmental conditions on human health.

## Data Availability Statement

The original contributions presented in the study are included in the article/supplementary material, further inquiries can be directed to the corresponding author.

## Ethics Statement

The studies involving human participants were reviewed and approved by Ethical Committee of the University of Zagreb Faculty of Pharmacy and Biochemistry. The patients/participants provided their written informed consent to participate in this study.

## Author Contributions

JD, AP, and MŽ conceived and designed research. AP and MB conducted sample collection. MŽ, AC, IA, AP, MB, NS, and OG preformed sample analyses. MŽ, DK, and SG preformed statistical analysis. JD, MŽ, AC, and IA wrote the manuscript. All authors read and approved the final version of the manuscript.

## Conflict of Interest

The authors declare that the research was conducted in the absence of any commercial or financial relationships that could be construed as a potential conflict of interest.

## Publisher’s Note

All claims expressed in this article are solely those of the authors and do not necessarily represent those of their affiliated organizations, or those of the publisher, the editors and the reviewers. Any product that may be evaluated in this article, or claim that may be made by its manufacturer, is not guaranteed or endorsed by the publisher.

## Footnotes

1
https://glycostore.org/
